# Quantitative analysis of EGR proteins binding to DNA: assessing additivity in both the binding site and the protein

**DOI:** 10.1186/1471-2105-6-176

**Published:** 2005-07-13

**Authors:** Jiajian Liu, Gary D Stormo

**Affiliations:** 1 Department of Genetics, Washington University School of Medicine, 660 S Euclid, Box 8232, St. Louis, MO 63110, U.S.A

## Abstract

**Background:**

Recognition codes for protein-DNA interactions typically assume that the interacting positions contribute additively to the binding energy. While this is known to not be precisely true, an additive model over the DNA positions can be a good approximation, at least for some proteins. Much less information is available about whether the protein positions contribute additively to the interaction.

**Results:**

Using EGR zinc finger proteins, we measure the binding affinity of six different variants of the protein to each of six different variants of the consensus binding site. Both the protein and binding site variants include single and double mutations that allow us to assess how well additive models can account for the data. For each protein and DNA alone we find that additive models are good approximations, but over the combined set of data there are context effects that limit their accuracy. However, a small modification to the purely additive model, with only three additional parameters, improves the fit significantly.

**Conclusion:**

The additive model holds very well for every DNA site and every protein included in this study, but clear context dependence in the interactions was detected. A simple modification to the independent model provides a better fit to the complete data.

## Background

Zinc finger proteins are the largest family of transcription factors in the human genome. The EGR sub-family of C2H2 zinc finger proteins has been extensively studied to determine the basis of DNA-protein binding specificity. The structure of the DNA-protein complex has been determined for the wild-type EGR1 (zif268) protein bound to its consensus site [1,2] and for several other variants of the interaction [3-5]. From the structure, the interaction appears very modular with each protein containing several zinc finger domains and each finger interacting with adjacent 3 base-pair (or overlapping 4 base-pair) segments of the binding site. Analysis of binding sites for this family of proteins suggested there were simple rules that relate the sequence of the zinc finger protein to its preferred binding site sequence [6], and that those rules could be used to design proteins with desired specificities [7,8]. Soon after, experimental techniques of *in vitro *randomization and selection were employed to greatly expand the collection of protein-DNA high affinity interactions [9-12]. Several reviews [4,13-18] have analyzed the protein-DNA crystal structures, summarized the results of the *in vitro *selection experiments, described rules for predicting high affinity protein-DNA interacting pairs and assessed the success of those rules for designing proteins to recognize particular sequences. Most of the recognition rules that have been developed are qualitative, specifying the amino acid and base-pair combinations that are preferred at each position in the binding sites [18]. Such rules can be effectively used to design proteins with preferred binding sites that are desired [19].

Despite the success of the qualitative recognition codes for designing proteins with desired preferred binding sites, the utility of such codes is still quite limited. If one compares the collection of known protein-DNA interacting pairs obtained in *in vitro *selection experiments, more than half of the fingers contain at least one amino acid/base-pair interaction that is not included in the code [20]. Furthermore, the code only predicts the preferred binding site for each protein sequence, or preferred protein for each DNA binding site. But it does not, by its qualitative nature, attempt to predict differences in affinities to similar sequences. Because all of these proteins bind with limited specificity, sites that are very similar to the preferred binding site can often bind with only slightly reduced affinity. Therefore predicting the quantitative binding specificities is important for a comprehensive view of their functions.

Several quantitative binding models have been developed, either specifically for the zinc finger proteins or for general protein-DNA interactions [20-26]. In many cases such codes can accurately predict the preferred binding sites as well as the qualitative codes, but the overall accuracy of the quantitative predictions is limited, undoubtedly for a combination of reasons. One reason is that there are limited data upon which to infer the model parameters using statistical approaches. Another reason is that many of the models are overly simplified, for instance assuming that each amino acid/base-pair contact is independent of any of the surrounding structure. We know, for instance, that the interactions of the protein and DNA are not completely additive [27,28], and it is also known that both intermolecular and intramolecular interactions contribute to protein-DNA recognition (24). But it has also been shown that models which are additive over the DNA positions can be a reasonably good approximations, at least for some proteins [29,30]. Most studies of additivity have focused on the DNA binding site, testing whether independent models for each base-pair fit the binding data well [29,31,32]. But equally important to the recognition codes is whether additivity holds within the protein. In one example from the EGR family, additivity within the protein was shown to be approximately additive (within 0.5 kcal) for one pair of mutated amino acids [33]. But very few studies have addressed the issue. Even though many variants of EGR family proteins have been used in SELEX and phage-display selection studies (see [20] for a summary), very few of the affinities have been quantified. Bulyk et al [28] did measure the affinity to each of 64 different binding sites for five different proteins, but the proteins were different at too many positions to be useful for determining additivity. One needs to have a set of single mutations and their double mutant combinations in order to determine whether the contributions to binding are independent or not. Several structural studies have highlighted the substantial rearrangements that can occur at the protein-DNA interface and can cause single amino acid or base-pair substitutions to influence the interactions at neighboring positions [3,15,34,35]. Such context effects may limit the predictive accuracy of simple recognition codes, although it is also possible that additivity can hold approximately even in the presence of such rearrangements. In the Mnt protein, a single amino acid change can alter the preferred binding site primarily at two adjacent positions, and more weakly over a longer distance [36,37]. Nevertheless, a complete quantitative analysis of the adjacent positions that were primarily affected showed that the interaction was largely additive for a wide variety of amino acid substitutions [30].

In this study we analyze the additivity of the interaction in both the DNA binding sites and in the interacting positions of the protein. We measure binding affinities for each of six different proteins, with single and double mutations compared to the wild-type protein, to each of six different DNA sites, also with single and double mutations from the wild-type binding site. We show that for any specific protein or DNA an additive model fits the data quite well. However, there are clear context effects such that no single interaction model fits all of the protein-DNA combinations. But only a small modification to the additive model, with just three additional parameters, improves the fit significantly.

## Results and discussion

**Figure 1:**
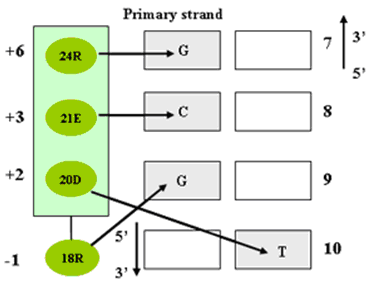
Amino acid-base contacts observed in co-crystal structures. The amino acid residues at -1, +2, +3, and +6 for zif268 are R, D, E and R, while the DNA bases at positions 7, 8, 9 and 10 for wild-type operator of zif268 are G, C, G and T.

Figure 1 diagrams the direct interactions between the amino acids of finger 1 of the zif268 protein with the bases of the consensus binding site as determined by X-ray crystallography [1,2]. In order to study the additivity of the interaction on the side of protein, we constructed wild-type zif268 and five mutants where mutations occur in finger one. These five mutants include two single mutants of zif268 at position -1 in which arginine (R18) (referred to as RE) was replaced by glutamine (Q) (referred to as QE) and aspartic acid (D) (referred to as DE), separately, one single mutant at position +3 where glutamic acid (E21) was mutated to asparagine (N) (referred to as RN), and two corresponding double mutants (referred as to QN and DN, respectively). The six DNA sites used for this study were chosen primarily based on the qualitative code that represents the correlations between amino acids located at different positions and the DNA bases that they specify [4,15,34]. Specifically, the anticipated base specificity for amino acids arginine, glutamine and aspartic acid at position -1 are G, A and C at position 9 in the DNA sequence, respectively. The favorable bases for amino acids glutamic acid and asparagine at position +3 are C and A at position 8. The oligos used to generate the six DNA sites are shown in Table 1. They share common sequences except for the DNA bases that are recognized by the amino acids at the position of +3 and -1 of finger 1, referred as CG, CA, CC, AG, AA, and AC, respectively. We measured the affinity of each of six proteins to each of six DNA sites, and we use these data to analyze the additivity in both the protein and the DNA binding sites.

**Table 1:** Oligos applied in this study. I: Synthesized DNA templates bearing either wild-type binding site (Zif_1) for zif268 or one of its variants (Zif_2 to Zif_6) used for generating DNA binding sites by PCR amplification, where KS-1 and SK-1 are two primers (low case). II: Oligos employed to construct five zif268 variants with QuickChange™ XL site-directed mutagenesis Kit (Stratagene) using pzif268 as a template.

I	Zif_1 tcgaggtcgacggtatcGCGTGGGCGCtccactagttctagagcggccgccac
	Zif_2 tcgaggtcgacggtatcGCGTGGGCACtccactagttctagagcggccgccac
	Zif_3 tcgaggtcgacggtatcGCGTGGGCCCtccactagttctagagcggccgccac
	Zif_4 tcgaggtcgacggtatcGCGTGGGAGCtccactagttctagagcggccgccac
	Zif_5 tcgaggtcgacggtatcGCGTGGGAACtccactagttctagagcggccgccac
	Zif_6 tcgaggtcgacggtatcGCGTGGGACCtccactagttctagagcggccgccac
	KS-1 tcgaggtcgacggtatc
	SK*-1 gtggcggccgctctagaact (SK-1 was fluorescent labeled with either FAM, HEX, TAMRA, ROX, or CY5)
II	18Q_plus 5' CGCCGCTTTTCTcagTCGGATGAGCTTACCCGCC
	18Q_minus 5' GGCGGGTAAGCTCATCCGActgAGAAAAGCGGCG
	18D_plus 5' CGCCGCTTTTCTgatTCGGATGAGCTTACCCGCC
	18D_minus 5' GGCGGGTAAGCTCATCCGAatcAGAAAAGCGGCG
	21N_plus 5' CGCCGCTTTTCTCGCTCGGATaacCTTACCCGCC
	21N_minus 5' GGCGGGTAAGgttATCCGAGCGAGAAAAGCGGCG
	18Q_21N_plus 5' CGCCGCTTTTCTcagTCGGATaacCTTACCCGCC
	18Q_21N_minus 5' GGCGGGTAAGgttATCCGActgAGAAAAGCGGCG
	18D_21N_plus 5' CGCCGCTTTTCTgatTCGGATaacCTTACCCGCC
	18D_21N_minus 5' GGCGGGTAAGgttATCCGAatcAGAAAAGCGGCG

For each protein we determined the relative affinity of each different binding site compared to the wild type site (CG) using the QuMFRA assay (Table 2). For the wild-type protein, the relative affinities of CA, CC, and AG to the reference site CG in this study are 0.27, 0.082 and 0.15, respectively. These data are in good agreement with the relative affinities previously determined by Miller and Pabo (0.21, 0.ll and 0.20, respectively [34]). Table 2 shows only the wild-type protein (RE) binds preferentially to the wild-type binding site (CG), all of the other proteins preferring a different binding site sequence. The range of affinities varies considerably between the different proteins. RE has about a 25-fold difference between the highest and lowest sites, while QE only varies by about 2-fold between the highest and lowest. We also measured the absolute binding affinity of each protein to one of the DNA binding sites with a Scatchard analysis (Table 3). The K_d _for wildtype zif268 binding to the DNA site CC is 3.0 × 10^-8 ^M, which converts to a K_d _for wildtype binding site CG of 2.5 × 10^-9 ^M. This value is almost the same as that determined by Hamilton et al (2.2 × 10^-9 ^M) [41] (previously reported values for this K_d _range from 0.04 to 6.5 nM, depending on the binding condition used [33]). No similar data exist for the other proteins in our collection. Combining the data from Tables 2 and 3, we derive the association constant of each protein for each different DNA sequence, which differ by over 300-fold between the highest and lowest affinities (Table 4).

**Table 2:** Relative binding constants for six DNA binding sites for wild-type of zif268 and its 5 derivatives, where wild-type operator of zif268 was used as the reference. Each data were obtained from 5 or more independent examinations, inside of parenthesis are the standard deviations.

DNA\Prot	RE(wt)	QE	DE	RN	QN	DN
CG(wt)	1	1	1	1	1	1
CA	0.27(0.06)	1.50(0.54)	1.16(0.49)	0.36(0.14)	0.49(0.19)	1.21(0.33)
CC	0.082(0.076)	2.17(0.91)	1.91(0.83)	0.41(0.23)	0.53(0.36)	2.61(0.59)
AG	0.15(0.10)	1.30(0.34)	1.48(0.56)	1.29(0.28)	4.45(2.64)	14.5(5.18)
AA	0.064(0.017)	1.36(0.48)	2.25(1.30)	0.68(0.28)	2.47(1.34)	4.02(1.56)
AC	0.041(0.045)	1.93(1.01)	3.08(0.45)	0.94(0.26)	2.78(0.80)	11.8(4.44)

**Table 3:** Experimental determined association constants (10^6^M^-1^) for individual indicated DNA binding site binding to its corresponding protein. Each value is the mean from 5 or more independent determinations and the standard deviations are shown in parenthesis.

DNA\Prot	RE(wt)	QE	DE	RN	QN	DN
CC	33(7)	6.4(1.7)	4.7(2.6)	33(14)		
AG					33(18)	17(6)

**Table 4:** Absolute *K*_*a*_(10^6^M^-1^) for six DNA binding sites and six variants of zif268, derived from the combination of Table 2 and Table 3.

DNA\Prot	RE	QE	DE	RN	QN	DN
CG	406	3.0	2.5	81	7.4	1.2
CA	109	4.5	2.8	30	3.5	1.4
CC	33	6.4	4.7	33	3.9	3.1
AG	63	3.9	3.6	105	33	17
AA	26	4.0	3.7	56	18	4.8
AC	16	5.7	5.5	77	21	14

From the binding data we can assess the additivity of the interaction for both the protein and the DNA. In a perfectly additive interaction the binding energy for each sequence would be the sum of the independent contributions at each position. For example, for any protein *j*, the binding energy to any DNA sequence *XY*, would be the sum of the interactions with base *X *and base *Y*:

Δ*G*_*j*_(*X*_8_*Y*_9_) = Δ*G*_*j*_(*X*_8_) + Δ*G*_*j*_(*Y*_9_).     (1)

The important assumption of the additive model is that the interaction energy at position 8, for example, doesn't depend on which base occurs at position 9. We do not expect additivity to hold precisely [30,27,28], but it can be a very good approximation, at least for some proteins [27,29]. Previously, studies of additivity have focused on whether the positions in the DNA binding site contribute independently to the binding of a particular protein. Using the data of Table 4 we can also determine whether the positions in the protein contribute additively to the binding of a particular DNA site. That is, we can reverse the symbols of equation 1 to refer to the binding of a particular DNA sequence, *i*, to a protein sequence *UV*:

Δ*G*_*i*_(*U*_-1_*V*_3_) = Δ*G*_*i*_(*U*_-1_) + Δ*G*_*i*_(*V*_3_).     (2)

Of course, we have not measured affinities to all possible DNA sequences or for all possible protein sequences, but because we have both single and double mutants in both the protein and the DNA, and have measured the binding affinities of all combinations, we can determine how well additivity holds on both sides, the DNA and the protein, at least for this limited set of variants.

We cannot actually measure the binding affinities to single positions because they always occur in some context. But we can find the "best fit" values for the independent interactions, and then determine how well the total data fits the additive model using those values. One method to obtain the best fit independent parameters is to apply multiple linear regression to the total data [31,32]. However, we have argued previously [29] that a better criterion is to minimize the difference in total free energy between the observed data and the model.



The  and  values are those obtained as the best fit parameters (those which minimize *M*) for each position assuming independence. The *ω *refers to either the protein or the DNA, and *α,β *refer to the residues at the two interacting positions. The first term inside the sum represents the probability that each particular residue sequence will be bound, and so weights the energy differences by their contribution to the total free energy of the system. As can be seen in the last form of the equation, *M *is the "mutual information" between the positions, the amount of total information content in the data that cannot be explained by the best independent model. We use log_2 _so that the mutual information is measured in bits.

Given the best fit independent parameters we can calculate the specificity information, *I*_*spec*_, of each position independently [42]. For example the specificity information for the protein or DNA *ω *at the first interacting position is



**Figure 2:**
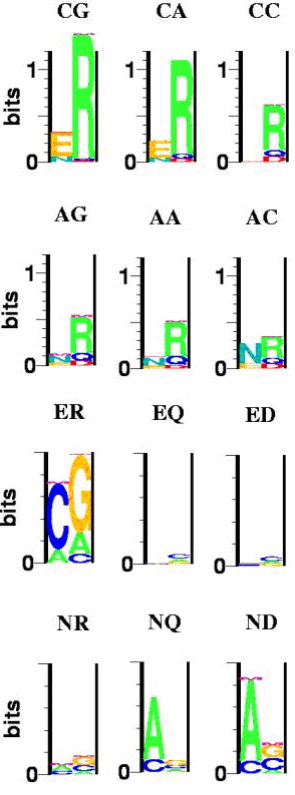
Sequence logos for each of six zinc finger proteins and the six DNA sites for which we have measured the affinity. M in each logo is the mutual information content in each interaction. The label at the top of each logo represents the DNA site (for the top two rows) or the protein (bottom two rows). The amino acid order is reversed so that they are lined up with the bases they contact. For example, the logo labeled "ER" shows the specificity for the RE (wild type) protein. In the lower six panels the maximum value on the y-axis is 0.5 bits.

*I*_*spec *_measures the amount of specificity in the interaction in bits; any non-specific protein or DNA would have *I*_*spec *_= 0. Figure 2 shows sequence logos [43] for each of the six proteins and the six DNA sequences for which we have measured the affinity. We have added the symbol "M" to each one which shows the amount of mutual information in each interaction [44,27,30]. That is the amount of total free energy, or specificity information, which is not captured by the best fit additive model. Half of the total mutual information is displayed above each position.

Several interesting results are evident in Figure 2. As stated above, the proteins vary considerably in their specificity, with RE (shown as "ER" in the figure) showing large discrimination between the different DNA sites, whereas QE and DE are fairly non-specific. The same holds for the different DNA sites, where CG is much more specific than CC or AC. It is interesting that every DNA site prefers R at position -1 of the protein, showing that it contributes to the total affinity of each protein as well as to the specificity of some proteins. The small degree of mutual information, the "M" in each logo, means that every interaction fits well with an additive model. Not only do the DNA positions contribute very additively, as has been shown previously for this family of proteins [29], but the contributions of the amino acids in the protein are also largely additive. The conclusion that additive models are good approximations to the true data holds for every DNA site and every protein included in the analysis. However, it is also true that there is not a single set of additive parameters that fit well for every case. This is consistent with the context effects previously noted for this family [15,34]. For example, R prefers to bind to G over A or C, but the magnitude of that preference is much larger if position +3 is an E instead of N. And an N at position +3 always prefers an A over C in the binding site, but that preference is much weaker with an R at position -1 than with a Q or D. Similarly, E at position +3 prefers a C very strongly in the context of an R, but is quite non-specific with either a Q or D at position -1. Similar effects, but of smaller magnitude, can be seen in the context effects of the DNA sites. These results show that additive models can be good approximations not only for the DNA sites in binding to any particular protein as has been seen before [29], but also for the proteins in binding to any particular DNA site. But the results also show that additivity for specific proteins and DNA sites is not sufficient to generate a general recognition code because context effects can still be important when both the DNA and protein can be variable. The small amounts of mutual information observed for any specific protein or DNA can be reinforced to give much larger amounts when measured over combinations of both components.

**Figure 3:**
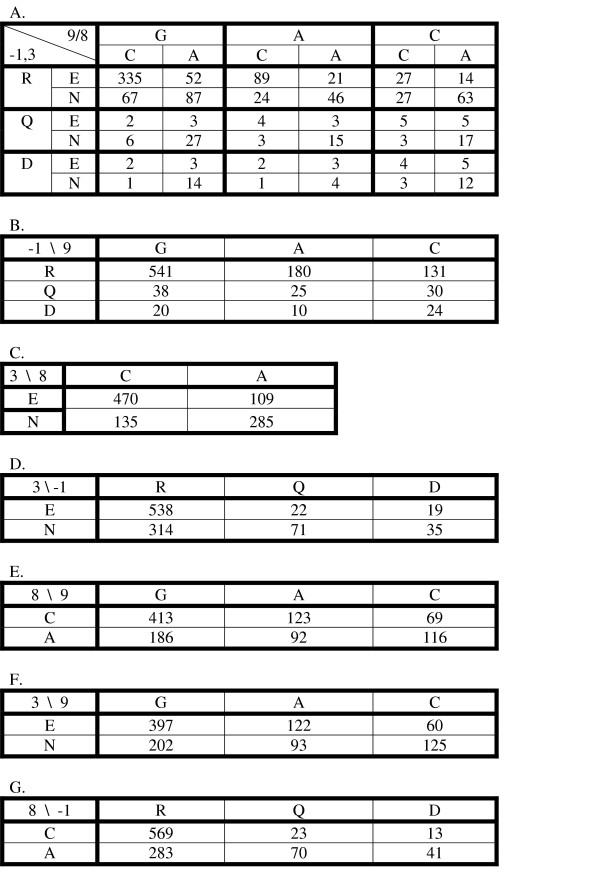
DNA binding specificities for six DNA sites for zif268 and its five derivatives. **A: **four-dimensional table representing binding specificities for all DNA sites and zinc finger proteins in this study. It is converted from Table 4 by normalization to a sum of 1000; **B: **2D table of combinations for the interaction of the amino acid at position -1 with the base-pair at position 9; **C: **2D table of combinations for the interaction of the amino acid at position +3 with the binding site position 8; **D: **2D table of combinations between amino acids at position -1 and +3; **E: **2D table of combinations between DNA bases at position 8 and 9; **F: **2D table of combinations between amino acid position 3 and base position 9; **G: **2D table of combinations between amino acid position -1 and base position 8.

To get a more detailed view of the dependencies in the data, it is useful to reformat it as in Figure 3A. Those data are the same as in Table 4 except that it has been normalized to a sum of 1000. In an experiment where every protein and DNA was equally available for binding, those elements in the table are 1000-times the probability of picking that particular combination from all of those in the bound state. The data are arranged in a four-dimensional (4D) table, with one dimension for each of the two positions in the protein and the two positions in the DNA. For example, the 335 at the RE-CG element of the table corresponds to the wild-type association constant of 406 from Table 4 after normalization. From the data in Figure 3A it is easy to obtain different lower dimensional views by summing over the other dimensions. For example, Figure 3B shows the 2D view of the interaction of the amino acid at position -1 with the base-pair at position 9 obtained by summing over all of the combinations of E,N at protein position +3 and C, A at binding site position 8 (inside the bold lines of Figure 3A). Similarly, Figure 3C shows a 2D view of the interaction between the amino acid at position +3 and the binding site position 8. Those two 2D views are orthogonal and together cover the 4D space of Figure 3A. We also show the remaining 2D views in Figures 3D–G. The pairs in Figure 3D,E and 3F,G are also orthogonal and together cover the 4D space of the data. If the binding interaction was completely additive, the true data of 3A could be calculated as the (renormalized) outer product of any pair of orthogonal matrices. Such predictions are not too bad, but demonstrate limitations of the additive model (see below).

Because the data in Figure 3 are in probabilities (if divided by 1000), the information specificity can be calculated more easily than in equation (4):

*I*_*spec*_(*α*) = log_2_*N*_*α *_- *H*_*α *_    (5)

where *α *is any of the positions or combination of positions, *H*_*α *_is the Shannon entropy of the data at those positions and *N*_*α *_is the number of entries in the data. For example, position -1 of the protein has three entries, R, Q and D, with overall probabilities of 0.852, 0.093 and 0.054, respectively, which gives *I*_*spec*_(- 1) = 0.84 bits. The upper half of Table 5 shows the specificity information for each of the positions (along the diagonal) as well as the specificity information for each of the pairs of positions (from the data shown in Figure 3). If the two positions contribute independently to the total specificity then the information for the paired positions is just the sum of the information at the each position. In this case the mutual information between the positions is the amount of information in the pair that exceeds the sum of the individual positions:

**Table 5:** Information for the position dependence. The diagonal is the specificity information for each of positions -1, 3, 8, and 9. The upper half of the matrix is the specificity information for each of the pairs of positions, and the lower half is the mutual information between pairs of positions.

**Position**	**-1**	**3**	**8**	**9**
**-1**	**0.84**	0.91	0.94	1.09
**3**	0.05	**0.02**	0.24	0.28
**8**	0.06	0.19	**0.03**	0.29
**9**	0.02	0.04	0.04	**0.22**

*M*(*α,β*) = *I*_*spec*_(*α,β*) - (*I*_*spec*_(*α*) + *I*_*spec*_(*β*))     (6)

Those values are shown in the lower half of Table 5. From the standard model of interaction between the DNA and protein we would expect there to be very little mutual information for any of the 2D datasets of Figure 3D–G, and that expectation is met. But we do expect high mutual information for the datasets in Figure 3B and 3C because those are the interacting positions. Just as we get high mutual information for positions that interact in RNA structures [44], we expect to see compensating changes between the amino acids and base-pairs that interact. That expectation is met for the combination of protein position +3 and base-pair position 8 (Figure 3C) where there is a clear preference for E binding to C and for N binding to A. In that case the mutual information is 0.19 bits, which is the main contribution to the total information of that pair, 0.24 bits. However, protein position -1 and base-pair position 9 also interact but show little mutual information because R is the preferred amino acid for each different DNA sequence and G is the preferred base-pair for each different protein. That pair has high specificity information, 1.09 bits, but it is very additive with only 0.02 bits of mutual information.

**Figure 4:**
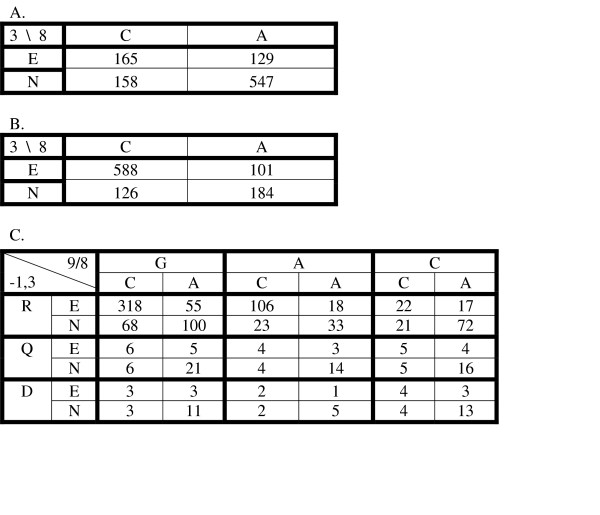
DNA binding specificities with the two component model. **A: **The 2D table of interactions for amino acid position 3 with base position 8 obtained from the data in Figure 3A for all cases except R with G or A (and normalized to a sum of 1000). **B: **The 2D table of interactions for amino acid position 3 with base position 8 for the cases with R and G or A (normalized to 1000). **C: **The predicted binding probabilities for the entire dataset using the two component model. The elements for the cases of R with G or A are obtained by the outer product of the matrix from **B **with the R/G,A elements of the matrix in Figure 3B. The rest of the elements are obtained from the outer product of **A **with the remaining elements of the matrix from Figure 3B.

**Figure 5:**
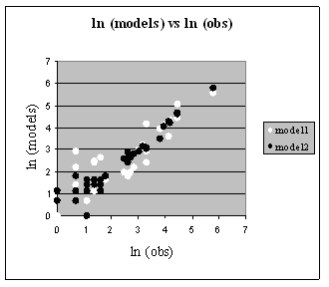
Scatter plot of the observed (Figure 3A) and predicted binding probabilities. Model2 is the two component model, so those points show the fit between Figure 3A and Figure 4C. Model1 is for the single component model obtained from the outer product of Figure 3B and Figure 3C (table of predicted probabilities not shown).

The total specificity information in the complete data of Figure 3A is 1.46 bits. The sum of the information for the interacting pairs, -1,9 and 3,8, is 1.33 bits, which shows that the complete specificity is reasonably well fit by assuming independent contributions from those interacting positions, as in most recognition code models [18]. If one predicts the complete data of Figure 3A as the outer-product of the matrices of Figure 3B and 3C (not shown), the correlation coefficient between the observed and predicted binding energies is 0.87 (Model 1 of Figure 5), similar to what had been observed previously for data in which only the DNA site had been varied [29]. While that result is reasonably good overall, examination of the complete data in Figure 3A identifies one clear source of context dependence between the interacting positions. When protein position -1 is R and the base-pair at position 9 is either G or A, there is a clear preference for the specific combination of E with C and a weak preference for N with A. But for all other combinations of positions -1 and 9, there is a strong preference for N with A, but very little preference for E. That is, the preference of E for C depends on the R with G or A combination being adjacent. In the structure of zif268 with the wild-type DNA there is no hydrogen bound between the position +3 E and the C base-pair, but rather it interacts with the backbone and with the neighboring R amino acid [2,1]. Various qualitative codes for the interactions of this protein family do not include E as an acceptable amino acid at position +3 [4,15]. But in the compilation of SELEX and phage-display results used by Benos *et al *[20], the combination of RE-CG was much more frequent than expected from the individual or pair occurrences (p-value less than 0.001). That is consistent with our result that in general E contributes little to the specificity of the binding site at position 8 except in the case where the adjacent interaction is R with G or A. Such context dependencies are not included in the simple recognition code models, but we can easily add that to the basic model. In Figure 4 we show two different specificity tables for the interaction of positions +3 and 8. Figure 4A represents the general case, and Figure 4B is for the special case of R with G or A at positions -1 and 9. If we now predict the complete data using these models, combined with the general model for positions -1 and 9 in Figure 3B, we obtain the values shown in Figure 4C. The specificity information of this data is 1.44 bits, showing that it models quite accurately the complete data. The correlation coefficient for those predicted binding energies with the measured energies is 0.96, a significant improvement over the model without the context dependent parameters (Model 2 of Figure 5). This improvement is at the cost of only three additional parameters due to the separation into two distinct classes depending on whether or not position -1 is an R that interacts with G or A. The completely additive model has 8 free parameters for the interaction of positions -1 and 9 (the 9 values in Figure 3B minus 1 for the total fixed sum) and 3 free parameters for the interaction of positions +3 and 8 (from the 4 values in Figure 3C). By separating the matrix of Figure 3C into two separate cases, shown in Figure 4A,B, we need 3 additional parameters in the model, for a total of 14. The model is used to predict data with 35 free values (the 36 elements of Figure 3A minus 1 for the fixed sum), so the additional parameters are only a small reduction in the degrees of freedom remaining to assess the fitness of the model.

The EGR family of proteins is an ideal case to study the effectiveness of a recognition code for protein-DNA interactions. The collection of crystal structures along with a large number of examples from selection experiments provides a wealth of information for determining the relationship between the protein sequence and the affinity for different DNA sequences. Simple qualitative models that predict the preferred interactions can be very effective and useful for designing new TFs [14,19]. Quantitative models, that predict relative binding affinities to multiple DNA sites, are more challenging but some success has been achieved by statistical approaches as well as by structure based approaches [20-26]. Most current models of this type assume independence of the contributions to binding between the positions in the interactions. In this work we show that additive models can be a good approximation for any particular EGR protein and also for binding to any particular DNA site; additivity holds well for both the DNA and protein side of the interaction. But we also show that there is not a universal set of parameters that work for all proteins or all DNA sites, rather there is context dependence in the interactions. However, at least in the cases studied here, a simple addition to the independent model that divides sites into two classes provides a much better fit. This holds promise that, even though additivity does not hold precisely, it may still be possible to determine an additive recognition code by identifying a small set of classes that cover the entire set of interactions. How many classes will be needed is unknown at this time. The 36 combinations in our study required only two classes to give a very good fit but this is still far from a comprehensive analysis. The total number of adjacent amino acid pairs is 400 and the number of di-nucleotide combinations is 16, so there are 6400 possible combinations of the two. Quantitative analyses that cover all possible combinations of even a single zinc finger are impossible at this time. But more thorough sampling of the space of high affinity interactions, followed by quantitative binding assays, will provide much valuable information regarding the nature of recognition codes. While a completely additive model for the interaction of the protein and DNA is not correct, it may be that only relatively minor modifications are needed to make significantly better predictions.

## Conclusion

By determining the binding affinities of single and double mutants in both the DNA binding site and in the protein we were able to assess the degree of additivity in both halves of the interaction. Although only a limited number of combinations were tested, we find that for every DNA sequence and for every protein sequence an additive model is a good approximation to the real binding data. However, when all of the data are considered together there are clear context effects that are not well fit by a single additive model. A slightly more complex model does provide a good fit to the observed data, suggesting that quite simple may still be employed to predict quantitative binding interactions of proteins with DNA. Further data are needed to determine how well these findings generalize to more variations and to other protein families.

## Methods

### Construction of wild-type zif268 DNA binding domain (DBD) and its variants

A plasmid containing the DNA binding domain of wild-type zif268 was obtained from Gendaq Limited [38]. The portion of zif268 cDNA encoding the three zinc-finger DBD (cDNA nucleotides 996–1262, amino acids 331–420) was amplified by PCR and subcloned into expression vector pET-28a-c(+) (Novagen) to create His-tagged fusion protein. The resulting construct, denoted pzif268, was verified by DNA sequencing. Five zif268 mutants with alterations in the base-contacting residues in finger one of zif268 DBD were constructed with QuikChange™ XL site-directed mutagenesis Kit (Stratagene) using pzif268 as a template: 3 single substitution mutants R18Q, R18D, E21N, and two double substitution mutants R18Q/E21N and R18D/E21N. The mutagenic primers containing the desired mutations used to create the five mutants are shown in Table 1. The resulting plasmids p18Q, p18D, p21N, p18Q21N and p18D21N were verified by DNA sequencing. Hereafter, the proteins are referred to by their amino acids at positions -1 and +3: RE (wild-type), QE, DE, RN, QN and DN.

### Expression and purification of His-tagged-zif268 fusion protein and its variants

*E. coli *BL21 cells bearing pzif268 or one of its derivatives were grown in 2xYT medium at 37°C with constant shaking at 250 rpm. IPTG was added to a final concentration of 1 mM when OD_600 _reached 0.6–1.0. Cells were harvested 3 hrs after IPTG induction by centrifugation at 4000 rpm for 20 min. The pellets were then resuspended in 15 ml of lysis buffer (50 mM Tris-HCl pH 8.0, 300 mM NaCl, 10 mM DDT and 1 tablet of protease inhibitor cocktail tablets (Roche) and lysed with sonication. The pellets were then separated by centrifugation at 6000 rpm for 20 min and insoluble material removed. The His-tagged fusion protein was purified with Ni-resin chromatography similar to those described previously [39]. The elutions were collected as 2 ml fractions. Fractions were analyzed on 12% SDS-PAGE gel, followed by silver staining. Finally the fractions were pooled and dialysed against dialysis buffer (30 mM Tris-HCl pH 8.0, 50 mM NaCl, 3 mM DTT) at 4°C, followed by concentration with a Centricon filter (Amicon) and kept at -80°C until usage. The protein concentration was determined with BioRad assay kit.

### Multiple quantitative fluorescence relative affinity (QuMFRA) assay to determine the relative binding constants

The relative binding constants of each protein to different binding sites were determined by the QuMFRA assay [27] with some modifications. Double-strand oligonucleotide binding sites used in this study were generated by PCR reactions. In each PCR reaction, a synthesized oligo containing either the wild-type binding site (zif1) of zif268 or one of its variants (Table 1) was used as template and the two primers are KS and SK (Table 1). The SK primer was labeled with one of the following four fluorophores: FAM, HEX, TAMRA, or ROX [27]. The PCR products were dissolved in TS buffer (10 mM Tris-HCl pH 8.0, 50 mM NaCl) after purification and precipitated with 1/10 vol of 3M NaAc and equal volume of isopropanol. The concentration of DNA was determined using a method similar to those as described previously [40].

The competitive binding assay [27] was performed by mixing 4 different fluorophore-labeled DNA binding sites with a certain amount of His-tagged zinc finger protein in 1x reaction buffer (30 mM Tris-HCl pH 8.0, 50 mM NaCl, 0.1 mg/ml BSA, 3 mM DTT, 20 uM ZnSO4, polydI-dC 5 ug/ml), in which the fluorophore-labeled zif1 served as an internal reference in each reaction. The reaction was equilibrated for 1 hr on ice before being electrophoresed on a 10% polyacrylamide gel. Each of 4 fluorophore-labeled PCR products was also loaded individually onto the same gel. After electrophoresis, the gels were scanned by a Typhoon Variable Scanner (Molecular Dynamics, Sunnyvale, CA) to obtain the fluorescent intensities of the separated bands (bound and unbound) at 4 different emission wavelengths using the same machine settings as employed by Man and Stormo [27]. For each separated band, the resultant fluorescence intensities at four emission wavelengths make up the output vector . Using the fluorescence intensities of the 4 individual fluorophore-labeled DNA at each emission wavelength we obtain the emission matrix *E *[27]. The input mixture of the 4 DNAs in each band, represented as the vector , were computed by a program developed for this study using the Gaussian elimination algorithm from the following relationship:



From the amount of each DNA in the bound and unbound bands of each lane, the relative binding affinity can be calculated by the following formula, where the wild-type binding site of zif268 (zif1) serves as the reference:

*K*_*b test*_/*K*_*b ref *_= *[P·D]*_*test*_*[D]*_*ref*_/*[D]*_*test*_*[P·D]*_*ref*_

*K*_*b test*_/*K*_*b ref *_= *I*_*P*-*Dtest*_*I*_*Dref*_/*I*_*Dtest*_*I*_*P*-*Dref*_

where *I*_*P*-*D *_and *I*_*D *_are the intensities of the specified DNAs in the bound and unbound bands, respectively.

### Determination of the absolute binding constant of a zinc finger protein to a binding site by Scatchard analysis

Scatchard analysis [41] was applied here to examine the absolute association constant, K_a_, of a zinc finger protein to a binding site. Specifically, a fixed amount of purified His-tagged zinc finger protein, [P]_total_, was mixed with increasing Cy5-labeled DNA generated by PCR reactions in 1x reaction buffer for 1 hr on ice. The bound and unbound DNA were separated by electrophoresis on a10% polyacrylamide gel, as above, and the gels were scanned by a Typhoon Variable Scanner using the excitation wavelength of 633 nm and emission wavelength of 670 nm. From the following relationship



it can be seen that the association constant for the particular combination of protein and DNA, K_*a*_(*P*,*D*), can be obtained from a plot of  at multiple DNA concentrations. At least five independent determinations were made for each protein.

## Authors' contributions

JL performed all of the experiments, which GS helped to design. Both authors contributed to the analysis of the data and the writing of the paper.
